# Modeling Bivariate Change in Individual Differences: Prospective Associations Between Personality and Life Satisfaction

**DOI:** 10.1037/pspp0000161

**Published:** 2017-09-18

**Authors:** Hilda Osafo Hounkpatin, Christopher J. Boyce, Graham Dunn, Alex M. Wood

**Affiliations:** 1 Primary Care and Population Sciences, Faculty of Medicine, University of Southampton, Southampton, United Kingdom; 2 Behavioural Science Centre, Stirling Management School, University of Stirling, and Manchester Centre for Health Psychology, School of Psychological Sciences, University of Manchester; 3 Centre for Biostatistics, Institute of Population Health, University of Manchester; 4 Behavioural Science Centre, Stirling Management School, University of Stirling, and Manchester Centre for Health Psychology, School of Psychological Sciences, University of Manchester

**Keywords:** personality, individual differences, life satisfaction, latent change score model, structural equation models

## Abstract

A number of structural equation models have been developed to examine change in 1 variable or the longitudinal association between 2 variables. The most common of these are the latent growth model, the autoregressive cross-lagged model, the autoregressive latent trajectory model, and the latent change score model. The authors first overview each of these models through evaluating their different assumptions surrounding the nature of change and how these assumptions may result in different data interpretations. They then, to elucidate these issues in an empirical example, examine the longitudinal association between personality traits and life satisfaction. In a representative Dutch sample (*N* = 8,320), with participants providing data on both personality and life satisfaction measures every 2 years over an 8-year period, the authors reproduce findings from previous research. However, some of the structural equation models overviewed have not previously been applied to the personality-life satisfaction relation. The extended empirical examination suggests intraindividual changes in life satisfaction predict subsequent intraindividual changes in personality traits. The availability of data sets with 3 or more assessment waves allows the application of more advanced structural equation models such as the autoregressive latent trajectory or the extended latent change score model, which accounts for the complex dynamic nature of change processes and allows stronger inferences on the nature of the association between variables. However, the choice of model should be determined by theories of change processes in the variables being studied.

An important endeavor in personality and social psychology is to understand individual differences in the developmental process (Mroczek & Spiro, 2003). The exploration of individual differences in the developmental process helps us to understand the change process and the dynamic relation between one or more psychological variables (Grimm, An, McArdle, Zonderman, & Resnick, 2012). However, there is a large amount of complexity to change processes. The life span development theory posits that there are two components to developmental change processes: intraindividual change (changes within individuals) and interindividual differences in intraindividual change (differences in intraindividual change between individuals) and that both of these components need to be considered to fully understand change (Baltes, Reese, & Nesselroade, 1977). This is because some people may not change at all over time, whereas others will but to varying degrees. Thus, it is an important research concern as to how such developmental change processes can be optimally modeled.

The choice of model should be determined by theories of the change processes in the variables being studied, and in the case of bivariate models, how two variables relate over time. Some psychological theories might predict, for example, simple unidirectional effects whereby initial levels of one variable may lead to change in a second variable, but initial levels of the second variable does not lead to change in the first variable. However, many psychological theories propose that there are often reciprocal effects between two variables, whereby initial levels of one variable predict subsequent changes in a second variable and initial levels of the second variable influence changes in the first variable. For example, individuals high on extraversion may experience increases in their well-being at a future time point. However, their level of extraversion may have itself been influenced by well-being at a previous time point (Soto, 2015). Furthermore, any reciprocal effects between two variables may become systematically stronger or weaker over time or be dependent upon environmental influences (Scarr & McCartney, 1983).

The presence of significant reciprocal effects indicate a dynamic relation between two variables. This dynamic relation becomes complex if, as predicted by many psychological theories, recent intraindividual changes in the first variable predict subsequent intraindividual changes in the second variable, and vice versa (Grimm, An, McArdle, Zonderman, & Resnick, 2012). For example, an individual who becomes more extraverted may then experience increases in well-being at a subsequent time point, yet increases in well-being at an earlier time point may have itself been an important contributor to increases in extraversion. The degree of intraindividual change in two reciprocally related variables may be the result of a proportional change process (whereby change in one variable is dependent upon immediately preceding levels of either variable) as well as due to a continuous developmental process (whereby there is a longer-term continuous change process; McArdle, 2009). For example, intraindividual change in well-being may be dependent upon levels of extraversion and well-being in the previous period (proportional change) as well as changes in well-being over time (continuous developmental process represented by mean-level changes in well-being).

One important advantage of studying how intraindividual changes in one variable predict subsequent intraindividual changes in a second variable is that it helps to overcome issues of omitted variable bias. There are often unchanging person-specific variables (such as ethnicity, genetic composition, or unobserved heterogeneous factors) that may be associated with either the first or second variable (Duckworth, Tsukayama, & May, 2010). Not accounting for such factors may confound any observed relation between two variables. The study of whether recent intraindividual changes, rather than recent levels, in one variable predict subsequent intraindividual changes in a second variable is therefore an important step in reducing omitted variable bias.

However, sometimes the models used to explore change processes do not always capture the level of complexity in the underlying developmental process. In part, this arises owing to unfamiliarity with appropriate modeling techniques to fully capture the developmental process. However, there are also data limitations. Ideally the study of whether intraindividual changes in one variable influence subsequent intraindividual changes in another variable requires data sets with three or more time-periods of data. With only two time-periods of data on each variable, it is also impossible to separate the proportional change (changes that are dependent on immediately preceding levels of each variable) from the continuous developmental processes (mean-level changes). Suitable statistical approaches that can model both these change processes are necessary to capture complexity of change. Historically, methods such as analysis of variance models or fixed-effects models were used to model individual differences in developmental processes. However, these models are not suitable due to restrictive assumptions regarding missing data, covariance structure of repeated measures (Hedeker & Gibbons, 2003) or, as in the case of fixed-effects models, do not account for measurement error.

In the current study, we overview four structural equation models that differentially model individual differences in the change process. Structural equation models are needed to account for measurement error in variables as well as model a complex dynamic relation between two variables over time. A number of structural equation models have now been developed that are increasingly being used to model developmental processes in one variable, as well as the longitudinal interplay between two or more developmental processes. We then explore their relevance theoretically and empirically to the study of the longitudinal association between personality traits and life satisfaction.

## Structural Equation Models of Change

Structural equation modeling (SEM) provides a framework for flexibly modeling change while simultaneously accounting for possible measurement error in the variables being studied. There are a number of different types of structural equation models and model selection may depend upon both the psychological theory being tested and the availability of data with sufficient time-periods. The most commonly used SEMs are the latent growth curve model (LGM; Curran, Obeidat, & Losardo, 2010), the latent autoregressive cross-lagged (ARCL) model (Jöreskog, 1979; Marsh & Grayson, 1994), the autoregressive latent trajectory (ALT) model (Bollen & Curran, 2006), and the latent change score (LCS) model (McArdle, 2009; Grimm, An, McArdle, Zonderman, & Resnick, 2012). Each of these models make different assumptions about the nature of change processes and therefore lead to different interpretations of the association between changes in two variables. Specifically, each model examines one or more of four processes of change and stability. The first process is the extent of stability in each variable. Stability in each variable is represented by an effect of previous levels of a variable on future levels of the same variable (an autoregressive effect). The second process is the dynamic relation between the two variables over time which is represented by an effect of prior levels of one variable on subsequent changes in a second variable (a cross-lagged effect). The third and fourth processes focus on intraindividual change. The third process is the continuous developmental process (mean-level change) in each variable that occurs over the entire available time period. The continuous developmental process may be due to genetic influences and everyday interactions with the environment. The fourth process is proportional change that is dependent upon immediately preceding levels of either variable. Proportional change accounts for variations in the rate of change with increasing levels of a variable.

**Figure 1 fig1:**
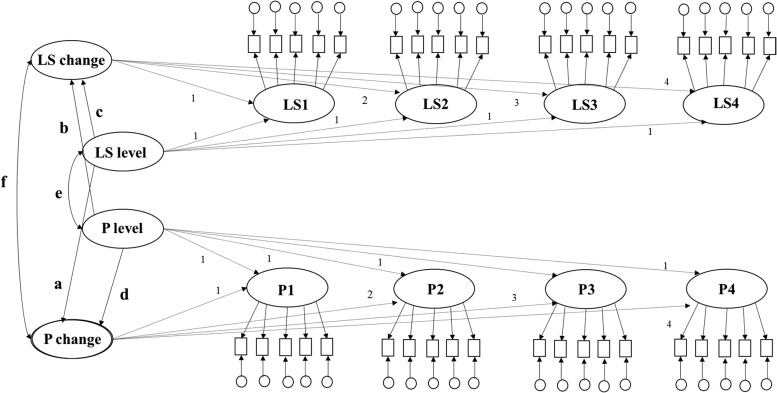
Bivariate latent growth model. Rectangles represent measured personality or life satisfaction variables at each time point. Ovals represent latent personality (P) and life satisfaction variables (LS) at each time point. LS level = initial life satisfaction level; P level = initial personality level; LS change = mean-level change in life satisfaction; P change = mean-level change in personality traits. Circles represent the measurement error present in each measured personality and life satisfaction variable respectively. Key paths are in bold format.

LGMs (Figure 1) and LCS models both examine developmental trajectories (indicated by an initial level term and a slope term representing the developmental process or mean-level change) in variables. Bivariate LGM and LCS models enable researchers to simultaneously examine if initial levels of one variable predict mean-level change in a second variable, and whether initial levels of the second variable predict mean-level change in the first variable. In the case of two-wave designs, a LCS is equivalent to a LGM, which allows developmental trajectories to vary across individuals but does not separate sources of change into a continuous developmental process (mean-level change) and proportional change (change that is dependent on the level of each variable at the immediately preceding time point). A limitation of LGMs and LCS models using data from two waves is that they are not fully prospective because they estimate initial levels and mean-level change scores using overlapping waves. In LGM and two-wave LCS models, both latent baseline levels and mean-level change scores are estimated using data from all available waves. In addition, in LGM and two-wave LCS models, any apparent association between initial levels of one variable and mean-level change in a second variable between Time 1 and Time 2 may in fact be due to other person-specific third variables (e.g., biological factors such as ethnicity), some of which may be unobserved (e.g., genetic composition) and therefore not easily adjusted for in model estimation (Duckworth, Tsukayama, & May, 2010).[Fig-anchor fig1]

**Figure 2 fig2:**
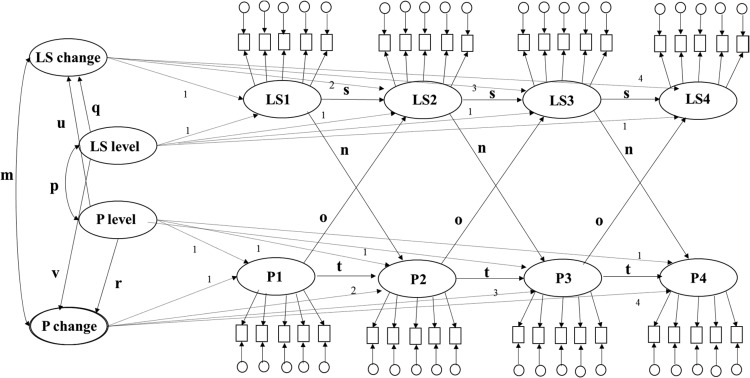
Bivariate latent change score (LCS) model, with mean-level and proportional changes. Rectangles represent measured personality or life satisfaction variables at each time point. Ovals represent latent personality (P) and life satisfaction variables (LS) at each time point. LS level = initial life satisfaction level; P level = initial personality level; LS change = mean-level change in life satisfaction; P change = mean-level change in personality traits. Circles represent the measurement error present in each measured personality and life satisfaction variable, respectively. ΔP_T2-T1_, ΔP_T3-T2,_ ΔP_T4-T3_ = proportional change in personality traits; ΔLS_T2-T1,_ ΔLS_T3-T2,_ ΔLS_T4-T3_ = proportional change in life satisfaction_._ Key paths of interest are in larger bold format.

With three or more wave designs, LCS models additionally offer the ability to divide the change process in each variable into a continuous developmental process and proportional change. This extended LCS model (see Figure 2) contains the developmental trajectory (initial level variable and slope variable representing mean-level change) present in LGMs but additionally contains change scores between consecutive waves. These between-wave change scores represent change that is proportional (McArdle, 2009) to the level of one or more variables at the preceding time point. These between-wave proportional change scores account for the fact that the extent to which changes in a variable across two assessment waves occurs is influenced by the level of that variable at the previous time point. Thus, the extended LCS model allows the study of whether intraindividual changes in one variable prospectively influence intraindividual changes in a second variable and vice versa. However, although allowing researchers to model complex developmental processes, the limitation of LCS models, including the extended LCS model, is that because of their complexity, they often provide a poorer fit on the data. Furthermore, the complexity of the models makes interpretation of paths between parameters somewhat difficult since the significance of some paths may be dependent on other paths in the model.[Fig-anchor fig2]

**Figure 3 fig3:**
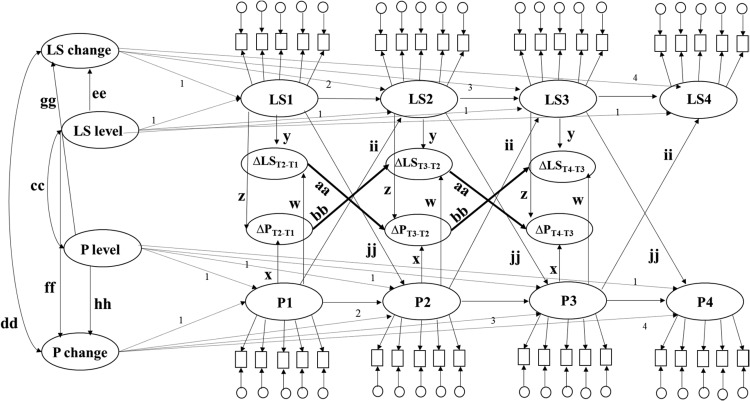
Bivariate latent autoregressive model. Rectangles represent measured personality or life satisfaction variables at each time point. Ovals represent latent personality (P) and life satisfaction variables (LS) at each time point. LS1 = initial life satisfaction level; P1 = initial personality level; LS2–LS4 = subsequent life satisfaction levels; P2–P4 = subsequent personality levels. Circles represent the measurement error present in each measured personality and life satisfaction variable, respectively.

In ARCL models (see Figure 3), change in each variable is assessed by regressing the latent score for each variable at Time 2 on the latent score of the same variable at Time 1. The ARCL model examines if baseline levels of a variable predict subsequent levels of the same variable (autoregressive effect). Bivariate ARCL models additionally examine if baseline levels of a variable predict subsequent levels of a second variable (cross-lagged effect). ARCL models only use data from the first wave (rather than all waves) to estimate baseline levels of a variable. Thus ARCL models are fully prospective and arguably better suited to test prospective associations between two variables than LGMs. However, unlike LGMs and LCS models, ARCL models do not explicitly model the developmental process. Mean-level change scores are needed to account for developmental processes (Barker, Rancourt, & Jelalian, 2014; Roberts, Walton, & Viechtbauer, 2006). The absence of mean-level change scores in ARCL models therefore may make such models relatively simplistic for modeling changes in developmental processes (Barker et al., 2014).[Fig-anchor fig3]

**Figure 4 fig4:**
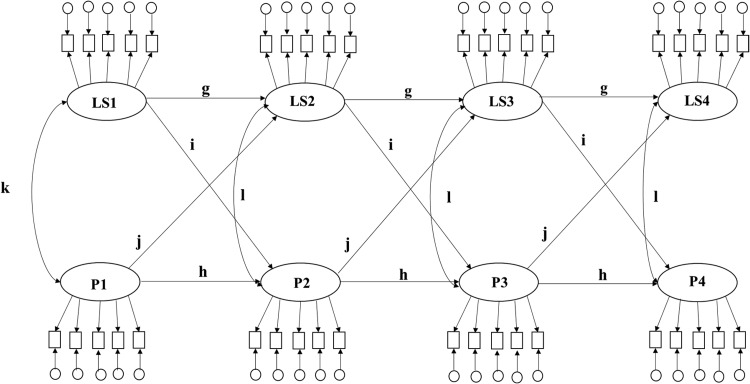
Bivariate autoregressive latent trajectory (ALT) models. Rectangles represent measured personality or life satisfaction variables at each time point. Ovals represent latent personality (P) and life satisfaction variables (LS) at each time point. LS level = initial life satisfaction level; P level = initial personality level; LS change = mean-level change in life satisfaction; P change = mean-level change in personality traits. Circles represent the measurement error present in each measured personality and life satisfaction variable, respectively. Key paths of interest are in bold format.

ALT models (see Figure 4) combine features of the LGM and ARCL models. ALT models estimate mean-level change (developmental change) in variables, while accounting for the fact that change in each variable is dependent on previous levels of each variable. In the case of bivariate ALT models this includes previous levels of the second variable. Although ALT models account for the fact that change in one variable is dependent on previous levels of either variable, change between consecutive waves is not explicitly estimated in the model. With ALT models, it is impossible to estimate how much change occurred between waves and how such ‘between-wave’ change in one variable relates to ‘between-wave’ change in a second variable. However, ALT models can be used to assess if reciprocal associations between initial levels of one variable and mean-level change in a second variable remain after accounting for initial levels of the second variable.[Fig-anchor fig4]

## Personality and Life Satisfaction

The relation between personality and life satisfaction is an example of a longitudinal relationship that has received considerable attention in recent years. Personality traits reflect individual differences in characteristic patterns of thoughts, feelings, and behavior (Roberts, Wood, & Caspi, 2008) and are thought to be one of the strongest predictors of life satisfaction (Steel, Schmidt, & Shultz, 2008). The application of structural equation modeling has helped develop the understanding of how these variables relate to one another.

Under the assumption that personality is largely fixed (Costa & McCrae, 1994; Srivastava, John, Gosling, & Potter, 2003) early research primarily used cross-sectional data to explore the relationship between personality and life satisfaction (e.g., DeNeve & Cooper, 1998). However, theoretical perspectives of change in personality suggests that personality in fact develops throughout an individual’s life (Srivastava, John, Gosling, & Potter, 2003) and this perspective has received substantial empirical support (Roberts, Walton, & Viechtbauer, 2006; Lucas & Donnellan, 2011). Such research has therefore ignited interest in understanding how changes in personality might relate to changes in life satisfaction, which is more readily agreed to change over an individual’s life (Baird, Lucas, & Donnellan, 2010). Although research has shown that an individual’s personality traits co-occur with changes in their life satisfaction levels (e.g., Boyce, Wood, & Powdthavee, 2013; Specht, Egloff, & Schmukle, 2013; Soto, 2015; Hounkpatin, Wood, Boyce, & Dunn, 2015), this relationship may arise either owing to a direct relationship, or indeed may be the product of a third variable.

Theoretically a cross-sectional association between personality and life satisfaction might arise owing to a direct relationship from personality to life satisfaction. Neuroticism, for example, is theoretically linked to life satisfaction via tendencies for an individual to experience negative and positive affect (Augustine & Larsen, 2015). Specifically, neuroticism is composed of facets such as anxiety, fear, and self-consciousness (Augustine & Larsen, 2015), which predispose an individual to experience negative affect. For this reason, highly neurotic individuals tend to appraise situations as stressful or threatening (Bolger & Schilling, 1991; Mroczek, Spiro, Griffin, & Neupert, 2006) and also react more negatively to challenging situations than less neurotic individuals (Bolger & Schilling, 1991; McCrae & Costa, 2003).

In contrast, extraversion is composed of facets such as excitement seeking and cheerfulness. Highly extraverted individuals tend to seek positive experiences, participate in more social activities (Srivastava, Angelo, & Vallereux, 2008), and respond more positively to situations and experiences (Lischetzke & Eid, 2006) than their introverted peers. These behaviors can help individuals feel more satisfied with life. Conversely, less extraverted individuals are more likely to experience low positive affect, which is related to a range of mental health conditions (Watson & Naragon-Gainey, 2010) and, as anhedonia, is a core component of depression (Dunn, 2012).

Theoretically other traits, such as agreeableness, conscientiousness, and openness are considered to have an indirect or instrumental relationship with life satisfaction in that changes to these traits might orientate individuals to situations that are likely to increase well-being (McCrae & Costa, 1991). For example, agreeable individuals are polite, considerate, and tend to cooperate with others better than less agreeable individuals. As a result, agreeable individuals are more likely to be liked by others, engage in more social activities, have a larger social network, and have strong stable personal relationships (Donnellan, Conger, & Bryant, 2004), which can contribute greatly to their life satisfaction (Powdthavee, 2008).

Individuals who are open to experiences tend to be broad-minded, artistic, and are able to appreciate, try, and enjoy new things and new ideas (Costa & McCrae, 1992). Open individuals are often concerned with enjoying experiences (Costa & McCrae, 1992) and therefore are more likely to engage in different activities (B. R. Little, Lecci, & Watkinson, 1992), which can help them enjoy their life. Furthermore, open individuals are also more likely to continuously seek opportunities to grow and develop further, which can lead to high levels of life satisfaction (Stephan, 2009).

Conscientious individuals are also more likely to be satisfied with their lives as they tend to be highly motivated, efficient, and thorough, which helps them avoid unemployment (Egan, Daly, Delaney, Boyce, & Wood, 2017) and generally have more satisfying jobs (Barrick, Mount, & Judge, 2001). Thus, increases in conscientiousness may result in higher life satisfaction through having a sense of greater achievement as well as financial rewards or promotions at work. Conscientiousness is also linked to better health, which may lead to higher life satisfaction (Israel et al., 2014).

Conversely, a reverse relationship may arise if an individual’s level of life satisfaction led to changes in their thoughts, feelings, and behavior, which resulted in changes in deep-seated personality traits (Roberts, Wood, & Caspi, 2008). For example, becoming less satisfied with life may cause one to start behaving in a socially withdrawn and cautious manner. Consistently behaving in a withdrawn manner can result in negative emotions, which subsequently lead to decreases in emotional stability, extraversion, agreeableness, conscientiousness, and openness over time (Soto, 2015). Similarly, becoming more satisfied with life may influence one to worry less, become more sociable and motivated, which, if consistent, would result in increases in emotional stability, extraversion, agreeableness, and openness over time.

## Understanding the Personality and Life Satisfaction Association Using Structural Equation Modeling

Some of the structural equation models outlined earlier have already been used to explore the relation between personality and life satisfaction. LCS models have now been used in several studies. However, to date only two waves of personality data have been explored. For example, Magee, Miller, and Heaven (2013) used the LCS model to show that initial levels of personality traits and mean-level changes in personality traits over a four year period predicted subsequent levels of life satisfaction in a representative sample of 11,104 Australian adults. Other studies have used the LCS with two waves of personality but, owing to more frequent availability of life satisfaction measures, incorporated life satisfaction using a latent growth model. Specht et al. (2013) carried out such an analysis using a representative sample of Germans (*N* = 14,718) who provided personality data twice over a four year period (during 2005 and 2009) and life satisfaction data yearly (2005, 2006, 2007, 2008, 2009), whereas Soto (2015) used a representative sample of 16,367 Australians who provided data on personality measures twice (during 2005 and 2009) over a 4-year period and data on life satisfaction yearly (2005, 2006, 2007, 2008, and 2009).

Overall, findings using the two-wave LCS models indicated that increases in emotional stability, extraversion, agreeableness, and conscientiousness across a 4-year period were significantly associated with mean-level changes in life satisfaction. Further, individuals with higher initial levels of life satisfaction subsequently experienced larger mean-level increases to their levels of emotional stability, agreeableness, and conscientiousness over a 4-year period compared to individuals who reported lower initial levels of life satisfaction. Together these findings suggest a reciprocal longitudinal association between personality traits and life satisfaction, whereby personality traits prospectively influence life satisfaction and life satisfaction prospectively influences personality traits.

A bivariate ARCL model (see Figure 3) has also been used to explore whether initial levels of a personality trait (life satisfaction) predict subsequent levels of life satisfaction (personality) after controlling for prior levels of the personality trait and prior life satisfaction levels. Soto (2015), in their study on Australian data (*N* = 16,367) and alongside the LCS model, also applied an ARCL model based on personality and life satisfaction measures in two time periods. The ARCL model indicated that individuals with higher initial levels of life satisfaction subsequently experienced higher levels of emotional stability, agreeableness, and conscientiousness over the 4-year period compared to individuals who reported lower initial levels of life satisfaction, and that individuals with higher initial levels of emotional stability, extraversion, agreeableness, and conscientious, subsequently experienced higher levels of life satisfaction than individuals who scored lower on these traits. Table 1 summarizes the characteristics of these models and findings from previous studies.[Table-anchor tbl1]

To the best of our knowledge, extended LCS models (which include proportional changes) (see Figure 2) and ALT models (see Figure 4) have not yet been specifically applied to study the longitudinal association between personality and life satisfaction. Both ALT and extended LCS models are useful as they can be used to examine if initial levels in a personality (or life satisfaction) variable influence within-person changes in life satisfaction (or personality) after accounting for autoregressive effects from prior levels of personality (or life satisfaction) and cross-lagged effects from prior levels of life satisfaction (or personality). In extended LCS models, the source of change may be segmented into continuous developmental processes and proportional change, which also allow the study of whether intraindividual changes in personality (or life satisfaction) predict subsequent intraindividual changes in life satisfaction (or personality).

In the current study, we examine prospective associations between personality traits and life satisfaction using bivariate LGM, latent ARCL models, ALT models, and extended LCS models. We use the Longitudinal Internet Studies for the Social Sciences (LISS) panel, administered by CentERdata (Tilburg University, the Netherlands) which contains personality and life satisfaction data every two years over an 8-year period, thus allowing us to apply the ALT and extended LCS models, which typically require three or more time-periods of data. We report on differences in results produced by the different models, discuss differences in interpretation of findings from each model, and consider the importance of using an appropriate model to study longitudinal change in developmental processes. Given the evidence in the literature of variability across demographic factors such as age (Magee et al., 2013) and gender (Durbin et al., 2016; Mueller et al., 2016) in trajectories of growth, we additionally explored whether age and gender moderated the longitudinal association between personality and life satisfaction. A previous study that used a LGM found increased emotional stability, extraversion, agreeableness, and conscientiousness were linked to increased life satisfaction, particularly for younger adults (Magee et al., 2013). However, to the best of our knowledge, no study has assessed whether age or gender moderate the association between personality and life satisfaction using LCS models containing data from three or more time points.

## Methods

This study did not require ethical approval as secondary anonymized data was used for the analyses.

### Participants and Procedure

Participants were part of the LISS panel, which is a representative random sample of the Dutch population. Households were randomly selected from municipal registers in 2007 and selected for inclusion in the panel if at least one member of the household was 18 years or older. Households that did not have a computer or Internet connection were provided with both and a €15 per hour incentive was provided to encourage long term participation (Knoef & deVos, 2009). Participants completed online surveys monthly. Surveys included questions on sociodemographics and psychological variables. An additional personality questionnaire was administered to all participants during May/August of 2009, 2011, 2013 and 2015.^1^ Our analytic sample consisted of 8,320 individuals who provided data on each item of both the life satisfaction and personality measures during at least one assessment wave. Mean age of the sample was 44.3 (*SD* = 15.81; age range: 10–95) and 53.8% were female. Of the 8,320 participants included in our study, 5,633 (68%), 5,312 (64%), 5,155 (62%), 4,781 (57%) participants responded to life satisfaction measures at Times 1, 2, 3, and 4, respectively, and 5,626 (68%), 5,298 (64%), 5,142 (62%), and 505 (6%) participants responded to personality traits at Times 1, 2, 3, and 4, respectively. Although considerably fewer participants responded to personality measures at Time 4, we included these data in our analyses because our analytic models use full information maximum likelihood estimation, which can use data on these variables from previous time periods to derive the most likely parameter estimates.

### Measures

#### Life satisfaction

The Satisfaction with Life Scale (Diener, Emmons, Larsen, & Griffin, 1985; Pavot & Diener, 1993) assessed satisfaction with life as a whole. This scale consisted of the following five items: “In most ways my life is close to my ideal,” “the conditions of my life are excellent,” “I am satisfied with my life,” “so far I have gotten the important things I want in life,” and “if I could live my life over, I would change almost nothing.” Respondents were asked to indicate how well each statement applied to them on a 7-point Likert scale, ranging from 1 (*strongly disagree*) to 7 (*strongly agree*). Higher scores represented higher life satisfaction. Cronbach’s alphas for the life satisfaction measure for our sample were .88, .89, .88, and .89 at Times 1, 2, 3, and 4, respectively. The test–retest reliability coefficient, as assessed by the intraclass correlation coefficient across the four time points for each item ranged from .55 to .58. This was calculated as the correlation between measures within a participant over time.

#### Personality

Personality was measured using the International Personality Item Pool (Goldberg, 1992; Goldberg et al., 2006) scale. Each personality trait was measured using 10 items. Respondents were asked how accurately each statement described them. Possible responses ranged from 1 (*very inaccurate*) to 5 (*very accurate*). Sample items included “I get stressed out easily” (neuroticism), “I’m the life of the party” (extraversion), “I have a rich vocabulary” (openness to experiences), “I feel little concern for others” (agreeableness; a reverse coded item), and “I’m always prepared” (conscientiousness). Reversely worded items were reverse-coded prior to generating five parcels containing two items for each personality trait. Items in parcels were consistent across time. Cronbach’s alphas for each personality trait during Times 1, 2, 3, and 4, respectively, were as follows: neuroticism—.88, .88, .88, and .90; extraversion—.86, .85, .86, and .85; openness—.74, .74, .73, and .76; agreeableness—.82, .81, .81, and .83; and conscientiousness—.80, .80, .79, and .83. Test-retest intraclass correlations across the four time periods ranged from .59 to .65, .64 to .69, .52 to .71, .53 to .59, and .56 to .66 for neuroticism, extraversion, openness, agreeableness, and conscientiousness, respectively. Means and standard deviations of each observed personality trait and life satisfaction measure are presented in Table 2.[Table-anchor tbl2]

### Analytical Strategy

#### Measurement model and measurement invariance

We first produced a separate measurement model for life satisfaction and separate models for each personality trait. The measured value of each life satisfaction item was specified to be the true value of life satisfaction and random measurement error. For each personality trait measure, parcels were formed each containing two items of the specified personality trait (Bagozzi & Heatherton, 1994; T. D. Little, Cunningham, Shahar, & Widaman, 2002). Each parcel was specified to be the true value of the personality trait and random measurement error. The variance in observed scores of each construct that was present in all assessment waves was isolated as the variance that is due to the underlying factor (Hoyle, 2012; i.e., the true score).

We further assessed strict measurement invariance (Bollen & Curran, 2006) to assess if the measurement model was consistent over time. To assess for measurement invariance in life satisfaction, we fitted a model containing four latent factors with five items each for life satisfaction at each assessment wave (2009, 2011, 2013, and 2015). We constrained the loading from corresponding items of life satisfaction to the latent life satisfaction factor to be equal across time. We also constrained the variances of the latent factors to be equal across time and constrained the error variances of the corresponding items (the degree of measurement error) to be equal across time. Finally, variances of the corresponding items of life satisfaction were specified to correlate across time to account for random measurement error. Similar models were fitted for each personality trait, using five parcels containing two items each as indicators of the specified personality trait at each assessment occasion.

#### Bivariate LGMs

Five bivariate LGMs were fit: one for each possible combination of personality trait with life satisfaction. Each LGM was specified as depicted in Figure 1. At each assessment wave, the true life satisfaction measure was represented by a latent score with five observed indicators (each indicator representing scores of one of the five life satisfaction items) and the true personality score was represented by a latent score with five observed indicators (each indicator representing the sum of two personality items comprising the parcel). A latent initial level and a latent slope variable (representing true mean-level change over time) were additionally estimated for life satisfaction and personality. Paths between the latent variables were then estimated as illustrated in Figure 1. The paths that were estimated using the LGM were: the concurrent correlation (represented by the correlation between initial levels of personality and initial levels of life satisfaction; path e in Figure 1), change correlation (represented by the correlation between personality slope and life satisfaction slope; path f in Figure 1), the prospective life satisfaction level effect (represented by a path from the initial level life satisfaction score to the latent slope personality score; path a in Figure 1), a prospective personality level effect (represented by a path from the initial level personality score to the latent life satisfaction slope; path b in Figure 1), the trait level-slope effect (represented by a path from the initial level personality score to the latent personality slope; path d in Figure 1), the life satisfaction level-slope effect (represented by a path from the initial level life satisfaction score to the latent life satisfaction slope; path c in Figure 1).

#### Bivariate latent autoregressive cross-lagged models

Five latent ARCL models were fit: one for each possible combination of personality trait with life satisfaction. Each ARCL model was specified as depicted in Figure 3. At each assessment wave, the true life satisfaction measure was represented by a latent score with five observed indicators (each indicator representing scores of one of the five life satisfaction items) and the true personality score was represented by a latent score with five observed indicators (each indicator representing the sum of two personality items comprising the parcel). For both life satisfaction and personality, the latent score at each assessment wave was specified to load on to the latent score at the immediately following assessment wave. Paths between the latent variables were estimated as shown in Figure 3. The paths that were estimated using the autoregressive models were: the concurrent correlation (represented by the correlation between the first latent personality score and the first latent life satisfaction score; path k in Figure 3), change correlation (represented by the correlation between subsequent latent personality scores and subsequent life satisfaction scores; path l in Figure 3), the prospective life satisfaction level effect (represented by a path from a latent life satisfaction score to the immediately following latent personality score; path i in Figure 3), a prospective personality level effect (represented by a path from a latent personality score to the immediately following latent life satisfaction score; path j in Figure 3), the trait stability (represented by a path from a latent personality score to the latent personality score at the immediately following assessment wave; path h in Figure 3), the life satisfaction stability (represented by a path from a latent life satisfaction score to the latent life satisfaction score at the immediately following assessment wave; path g in Figure 3).

#### Bivariate autoregressive latent trajectory models

Five ALT models were fit: one for each possible combination of personality trait with life satisfaction. Each ARCL model was specified as depicted in Figure 4. At each assessment wave, the true life satisfaction measure was represented by a latent score with five observed indicators (each indicator representing scores of one of the five life satisfaction items) and the true personality score was represented by a latent score with five observed indicators (each indicator representing the sum of two personality items comprising the parcel). For both life satisfaction and personality, the latent score at each assessment wave was specified to load on to the latent score at the immediately following assessment wave. A latent initial level and a latent slope variable (representing true mean-level change over time) were additionally estimated for life satisfaction and personality. Paths between the latent variables were estimated as shown in Figure 4. The paths that were estimated using the ALT models were: the concurrent correlation (represented by the correlation between initial levels of personality and initial levels of life satisfaction, path p in Figure 4), change correlation (represented by the correlation between personality slope and life satisfaction slope, path m in Figure 4), the prospective life satisfaction level effect (represented by a path from the initial level life satisfaction score to the latent ‘slope’ personality score, path v in Figure 4), a prospective personality level effect (represented by a path from the initial level personality score to the latent life satisfaction ‘slope,’ path u in Figure 4), the trait level-slope effect (represented by a path from the initial level personality score to the latent personality slope, path r in Figure 4), the life satisfaction level-slope effect (represented by a path from the initial level life satisfaction score to the latent life satisfaction slope, path q in Figure 4), the trait stability (represented by a path from a latent personality score to the latent personality score at the immediately following assessment wave, path t in Figure 4), the life satisfaction stability (represented by a path from a latent life satisfaction score to the latent life satisfaction score at the immediately following assessment wave, path s in Figure 4).

#### Bivariate latent change score models

Five extended LCS models (McArdle, 2009) were estimated: one for each possible combination of personality trait with life satisfaction. Each LCS model was specified as shown in Figure 2. At each assessment wave, the true life satisfaction measure was represented by a latent score with five observed indicators (each indicator representing scores of the five life satisfaction items) and the true personality score was represented by a latent score with five observed indicators (each indicator representing the sum of two personality items comprising the parcel). A latent initial level and a latent slope variable (representing mean-level change over time) were also estimated for life satisfaction and personality. In addition, each LCS model contained proportional latent change scores between consecutive waves of personality (ΔP_T2-T1,_ ΔP_T3-T2,_ ΔP_T4-T3_ in Figure 2) and life satisfaction (ΔLS_T2-T1,_ ΔLS_T3-T2,_ ΔLS_T4-T3_ in Figure 2), which accounted for the influence of immediately preceding levels of personality and life satisfaction on changes in personality and life satisfaction, respectively. Paths were then introduced to allow: the latent slope personality score to be influenced by the initial level life satisfaction score (the prospective life satisfaction level effect; path *ff* in Figure 2) and the initial level personality score (trait-level slope effect; path hh in Figure 2), the latent slope life satisfaction score to be influenced by the initial level personality score (the prospective personality trait level effect; path gg in Figure 2) and initial level life satisfaction score (life satisfaction-level effect; path ee in Figure 2). Life satisfaction levels were specified to be influenced by levels of life satisfaction at the immediately preceding assessment wave (autoregressive effect), and levels of personality at the immediately preceding assessment wave (cross-lagged effect). Personality levels were specified to be influenced by levels of personality at the immediately preceding assessment wave (autoregressive effect), and levels of life satisfaction at the immediately preceding assessment wave (cross-lagged effect). Each proportional latent change score for personality was then specified to be influenced by immediately preceding levels of life satisfaction (path z in Figure 2) and immediately preceding levels of personality (path x in Figure 2), and each proportional latent change score for life satisfaction was specified to be influenced by immediately preceding levels of personality (path w in Figure 2) and immediately preceding levels of life satisfaction path y in Figure 2). A path between initial levels of personality and initial levels of life satisfaction (representing concurrent correlations; path cc in Figure 2) and a path between personality trait slope and life satisfaction slope (representing change correlations; path dd in Figure 2) were also estimated. Finally, paths were specified between proportional change scores in personality and subsequent proportional change scores in life satisfaction (path bb in Figure 2) and between proportional change scores in life satisfaction and subsequent personality change scores in personality (path aa in Figure 2).

For each LGM, ARCL, ALT, and LCS model, error variances of observed indicators of personality were constrained to be equal over time, error variances of observed indicators of life satisfaction were constrained to be equal over time, and indicators of personality and life satisfaction over time were allowed to correlate. Each LGM, ARCL, ALT, and LCS model additionally controlled for the effects of age and gender. We repeated each LCS model including interaction terms for initial levels of personality with age and gender (separately) and initial levels of life satisfaction with age and gender (separately) and initial levels of life satisfaction with age and gender (separately). We were unable to control for ethnicity as this was missing for a large number of individuals (92.3% of total sample) and where data for this variable was not missing it was poorly coded. All models were fitted using Mplus Version 5 (Muthén & Muthén, 1998-2007). The fit of each model was evaluated using fit criteria suggested by Hu and Bentler (1999); a model with comparative fit index (CFI) > .95, root mean squared approximation index (RMSEA) < .06 and standardized root-mean-square residual (SRMR) < .08 is considered to fit the data well. Each model was estimated based on the full information maximum likelihood estimator which accounts for missing data by using all available data and borrowing information about the correlation between variables in complete cases to produce the most likely estimates of the parameters of interest (Allison, 2012).

## Results

### Measurement Model and Measurement Invariance

Each of our measurement models for personality traits and our measurement model for life satisfaction produced satisfactory fit (CFI > .90, RMSEA < .06, SRMR < .06). The models assessing strict measurement invariance produced good fit (CFI > .90, RMSEA < .06, SRMR < .06), indicating our measurement models were consistent over time.

### Analytical Models

We fitted four different types of structural equation models to assess the longitudinal association between personality traits and life satisfaction. We additionally fitted single variable models for each personality trait and life satisfaction, each of which indicated good fit and significant change variance. Each type of model produced good fit on the data but produced different results. Each model indicated significant cross-sectional association between personality and life satisfaction. Higher initial levels of life satisfaction were associated with higher initial levels of extraversion, openness, agreeableness, conscientiousness, and lower initial levels of neuroticism. Further results of each type of model are reported below and presented in Tables 3, 4, 5, and 6. In Tables 3–6, each estimated path is labeled with the same letters as their respective paths in Figures 1–4.[Table-anchor tbl3][Table-anchor tbl4][Table-anchor tbl5][Table-anchor tbl6]

### Bivariate LGMs

Bivariate LGMs (see Figure 1) estimate overall developmental trajectories, represented by initial levels and slope (mean-level change), of personality traits and life satisfaction using data from all assessment waves. The LGM assesses whether developmental trajectories of personality traits relate to developmental trajectories of life satisfaction. The LGM also allows the trajectories to vary across individuals and estimates how these interindividual differences in personality traits and life satisfaction levels predict intraindividual changes in both personality traits and life satisfaction. However, the LGM does not model stability in personality traits (or life satisfaction) measures over time and does not allow the researcher to assess the direction of the association between intraindividual changes in personality and life satisfaction.

Our LGM indicated an increase in mean-level change in life satisfaction over the 8-year period was associated with increases in mean-level change in extraversion, agreeableness, conscientiousness, and a decrease in mean-level change in neuroticism over the 8-year period (Table 3, path f). Our LGM also indicated that higher initial levels of extraversion, openness, agreeableness, and neuroticism predicted an increase in mean-level change in life satisfaction (Table 3, path b, *p* < 0.05). Higher initial levels of life satisfaction predicted increases in mean-level change in neuroticism, extraversion, openness, and conscientiousness (Table 3, path a, *p* <0.05). In summary, the LGMs suggested individual differences in life satisfaction predicted mean-level changes in personality traits and individual differences in personality traits predicted mean-level changes in life satisfaction.

### Bivariate Latent ARCL Models

Bivariate ARCL models (see Figure 3) estimates (latent) initial levels of personality traits and life satisfaction. The bivariate ARCL model then assesses whether initial levels of personality and life satisfaction predict subsequent changes (represented by regressing subsequent level on prior level) in personality and life satisfaction, after controlling for autoregressive effects. In this way the bivariate ARCL model, unlike the LGM, estimates stability in personality traits over time and stability in life satisfaction measures over time. However, the bivariate ARCL model does not model mean-level changes in personality traits and life satisfaction over time.

Our bivariate ARCL models indicated higher subsequent levels of life satisfaction were associated with higher subsequent levels of extraversion, openness, agreeableness, conscientiousness and lower subsequent levels of neuroticism (Table 4, path l). Each bivariate ARCL model also indicated that levels of personality traits at any given time were strongly positively associated with personality levels at a previous time point (Table 4, path h), and levels of life satisfaction at any given time were strongly positively associated with life satisfaction levels at a previous time point (Table 4, path g; autoregressive effects). Finally, each bivariate ARCL model suggested that lower initial levels of neuroticism and higher initial levels of extraversion, agreeableness, and conscientiousness predicted higher subsequent life satisfaction levels (Table 4, paths j). Initial levels of life satisfaction did not predict subsequent personality trait level, except for decreases in openness (Table 4, paths i). In summary, the ARCL models suggested individual differences in life satisfaction did not predict subsequent levels of personality traits but individual differences in personality traits predicted subsequent levels of life satisfaction, after accounting for autoregressive effects.

### Bivariate ALT model

The ALT model (see Figure 4) combines characteristics of the ARCL model and LGM. The ALT model allows personality trait level at one time point to be predicted from mean-level change (developmental process represented by slope variable) in personality as well as from levels of personality at a previous time point (represented by regressing subsequent level on prior level). Similarly, the ALT model allows life satisfaction level at one time point to be predicted from mean-level change in life satisfaction as well as from levels of life satisfaction at a previous time point. The ALT model also estimates whether initial personality trait level prospectively influences mean-level change in life satisfaction and mean-level change in personality and whether initial life satisfaction level prospectively influences mean-level change in personality traits and mean-level change in life satisfaction.

Our ALT models indicated an increase in mean-level change in life satisfaction was significantly associated with increases in mean-level changes in extraversion, openness, agreeableness, conscientiousness, and a decrease in mean-level change in neuroticism (Table 5, path m). Each ALT model further indicated the levels of life satisfaction at any given time were predicted by prior life satisfaction levels (Table 5, path s) and levels of personality traits at any given time were predicted by prior personality levels (Table 5, path t). Higher initial levels of life satisfaction levels prospectively predicted a decrease in mean-level change in life satisfaction (path q in Table 5) and higher initial levels of neuroticism and openness prospectively predicted a decrease in mean-level change in neuroticism and openness, respectively (path r in Table 5). Lower initial levels of neuroticism and higher initial levels of extraversion, openness, and agreeableness prospectively predicted an increase in mean-level change in life satisfaction (path u, Table 5). Higher initial levels of life satisfaction prospectively predicted a decrease in mean-level change in neuroticism and increases in mean-level changes in extraversion, openness, agreeableness, and conscientiousness (path v, Table 5). In summary, the ALT model indicated individual differences in personality predicted mean-level change in life satisfaction and individual differences in life satisfaction predicted mean-level changes in personality traits, after controlling for autoregressive effects.

### Bivariate LCS Models Specifying Associations Between Proportional Changes in Personality and Proportional Changes in Life Satisfaction

LCS models estimate initial levels and mean-level changes (represented by the slope variables) in personality traits and life satisfaction and also estimates proportional changes between consecutive waves. The LCS specifies that each proportional change score for personality is influenced by the mean-level change score in personality as well as previous levels of personality, previous levels of life satisfaction and previous proportional changes in life satisfaction. Like the LGM, the LCS model can estimate whether personality trait level influences mean-level changes in life satisfaction and personality, and whether life satisfaction levels influence mean-level changes in personality traits and life satisfaction. In addition to that, the LCS model estimates whether proportional changes in personality traits influence subsequent proportional changes in life satisfaction and whether proportional changes in life satisfaction influence subsequent proportional changes in personality traits.

Our bivariate LCS models indicated higher initial levels of life satisfaction predicted a decrease in mean-level change in neuroticism over the 8-year period (Table 6, path ff) and increases in mean-level changes in openness, agreeableness, and conscientiousness over the 8-year period (Table 6, path ff). Higher initial levels of extraversion, openness, agreeableness, and conscientiousness predicted an increase in mean-level change in life satisfaction over the 8-year period (Table 6, path gg). Higher initial levels of neuroticism predicted a decrease in mean-level change in life satisfaction over the 8-year period (Table 6, path gg). Mean-level changes in personality traits (except for conscientiousness) were significantly associated with mean-level changes in life satisfaction (Table 6, path dd) even after accounting for the association between proportional changes in life satisfaction and proportional changes in personality traits. Increases in proportional changes in openness and conscientiousness (but not the remaining traits) predicted an increase in subsequent proportional change in life satisfaction (Table 6, path bb). An increase in proportional change in life satisfaction predicted a subsequent decrease in proportional change in neuroticism and increases in proportional changes in extraversion, openness, agreeableness, and conscientiousness (Table 6, path aa). In summary, the LCS models suggested that individual differences in personality traits predict mean-level change in life satisfaction, individual differences in life satisfaction predict mean-level changes in personality traits, proportional changes in some personality traits predict subsequent proportional change in life satisfaction and proportional change in life satisfaction predicted subsequent proportional changes in each of the personality traits.

Separate LCS models containing an interaction term for initial levels of personality traits with age and an interaction term for initial levels of life satisfaction with age indicated age interacted with initial levels of life satisfaction to predict mean-level change in life satisfaction (β = 0.01, *p* = .006). This model suggested higher initial life satisfaction was associated with a mean-level increase in life satisfaction for older individuals but not for younger individuals. A separate model indicated age interacted with initial levels of conscientiousness to predict mean-level change in conscientiousness (β = −0.01, *p* < .001), suggesting higher initial levels of conscientiousness predicted a mean-level decrease in conscientiousness, particularly for older adults. A separate model indicated age interacted with initial levels of agreeableness to predict mean-level change in agreeableness (β = −0.01, *p* < .001). This model suggested higher initial levels of agreeableness predicted a mean-level decrease in agreeableness, particularly for older adults. An LCS model containing an interaction term for initial levels of agreeableness with gender and an interaction term for initial levels of life satisfaction with gender indicated gender interacted with initial levels of agreeableness to predict mean-level change in agreeableness (β = −0.05, *p* = .018) and life satisfaction (β = −0.05, *p* < .001). This model suggested higher initial levels of agreeableness was associated with a mean-level decrease in agreeableness, particularly for females, and a mean-level increase in life satisfaction, particularly for males. Finally, a separate model containing an interaction term for initial levels of extraversion with gender and an interaction term for initial levels of life satisfaction with gender indicated gender interacted with initial levels of extraversion to predict mean-level change in life satisfaction (β = −0.03, *p* = .027). This model suggested higher initial levels of extraversion was associated with a mean-level increase in life satisfaction, particularly for males.

## Discussion

Different types of structural equation models may be used to examine developmental processes. The most common of these models are the LGM, the ARCL model, the ALT model, and the LCS model. The choice of model depends primarily on the psychological theory being tested. However, data considerations are also an important concern and not having variables observed over sufficient time-points can limit the possibility of establishing and separating both the continuous developmental process (mean-level change) and proportional change (change that is dependent on the level of each variable at the immediately preceding time point). In this article, we explored each of these models and demonstrate how each of these models may result in different interpretations of the longitudinal association between personality traits and life satisfaction. We found that our LGMs, ALT models, and LCS models indicated a reciprocal dynamic relation between prior levels of personality traits and subsequent mean-level changes in life satisfaction and prior levels of life satisfaction and subsequent mean-level changes in personality traits. However, the ARCL models suggested that initial levels of personality traits predicted subsequent levels in life satisfaction but initial levels of life satisfaction did not predict subsequent levels of personality traits.

### Implications for the Longitudinal Association Between Personality and Life Satisfaction

Previous research has shown that changes in personality traits co-occur with changes in life satisfaction (e.g., Magee et al., 2013; Specht et al., 2013; Soto, 2015). These studies have also indicated an association between initial levels of personality traits and subsequent changes in life satisfaction (Magee et al., 2013; Soto, 2015), initial levels of life satisfaction and subsequent changes in personality traits (Specht et al., 2013; Soto, 2015), or both (Soto, 2015). Together these studies suggest that highly emotionally stable, extraverted, agreeable, and conscientious individuals are more likely to experience subsequent mean-level increases in life satisfaction over time, and individuals who are more satisfied with their lives are more likely to experience subsequent mean-level increases in emotional stability, agreeableness, and conscientiousness. Thus these findings support psychological theories that propose a reciprocal longitudinal association between personality and life satisfaction at the between-person level.

The findings from the LGMs, ALT models, and LCS models in the current study are consistent with previous studies and suggest a concurrent association between changes in personality and life satisfaction and a reciprocal longitudinal association between personality and life satisfaction at the between-person level. Similar to previous studies, the LGMs here suggests that this reciprocal association exists between initial levels of and overall changes in personality and life satisfaction. This overall change may include developmental processes, as well as change from other processes. The ALT models and LCS models further suggest that this dynamic relation exists between initial levels of and constant developmental change processes in personality traits and life satisfaction.

The LCS models, however, indicated a more complex dynamic relation between personality traits and life satisfaction than either the LGMs or ALT models. The LCS models indicated a unidirectional longitudinal association between personality and life satisfaction also exists at the within-person level. Individuals who experienced increases to their life satisfaction subsequently experienced increases in emotional stability, extraversion, openness, and agreeableness. This provides evidence that the association between individual differences in initial levels of personality traits and mean-level change in life satisfaction is not confounded by other (time-invariant) person-specific variables. Our LCS models did not support theories of within-person reciprocal longitudinal association between personality and life satisfaction but rather indicated the longitudinal association between personality and life satisfaction may be a result of changes in life satisfaction predicting subsequent changes in personality traits.

There was also evidence that age and gender interacted with the developmental process, indicating that older people with higher levels of agreeableness and conscientiousness were more likely, than younger people with similar levels of each trait, to experience mean-level decreases in agreeableness and conscientiousness over time. Extraverted males were also more likely than extraverted females to experience a mean-level increase in life satisfaction over time. These findings are broadly consistent with previous research highlighting age and gender moderation effects in growth trajectories (e.g., Durbin et al., 2016).

Although we did not find evidence to support an association between initial life satisfaction levels and subsequent personality levels using the ARCL, previous studies have found significant associations between initial levels of personality and subsequent levels of life satisfaction, and vice versa. There are a number of possible reasons for the discrepancies between our results and that of previous research. The differences may be due to slight differences in methodologies. For example, the use of two rather than four waves, differences in the time intervals between repeat assessments, differences in the number and formation of parcels used as indicators of personality and life satisfaction, or slight differences in specification of the ARCL models across studies. It is also possible that there were stronger autoregressive effects between personality traits in our cohort. Alternatively, the process of personality and life satisfaction may be more complex in our specific sample, which may require the specification of both mean-level and proportional changes in order for the association between individual differences in life satisfaction and subsequent levels of personality to manifest.

### Implications for the Study of Individual Differences in Change

The current study highlights that each of these commonly used structural equation models examine different aspects of change. Each model makes different assumptions regarding the nature of change and, in the case of bivariate models, the nature of the longitudinal association between two variables. We demonstrate that it is important to use a model that fully captures the dynamic process of change predicted theoretically. With the increasing availability of repeated measurements across multiple time periods, it is possible to apply more advanced models of change and in doing so allows a better understanding of the nature of the dynamic relation between two psychological variables. It is likely that the LGM and ARCL models are less suitable to model the longitudinal association between developmental change processes as they do not fully capture the developmental processes in variables. For example, the LGM models intraindividual changes in each construct (personality and life satisfaction, in our example) as a single overall trajectory that represents mean-level change in the variable. The LGM therefore makes the assumption that each variable changes in a continuous and steady fashion and does not explicitly account for the fact that changes in each variable are influenced by prior levels of the same variable, as well as prior levels of the second variable. In contrast, the ARCL captures autoregressive effects for each variable (prior levels of the variable influencing subsequent levels of the same variable) as well as cross-lagged effects between the two variables (prior levels of one variable influencing subsequent levels of the second variable) but does not estimate mean-level change scores, which represent the developmental trajectory (Roberts, Walton, & Viechtbauer, 2006).

The ALT model more adequately captures dynamic change as it examines mean-level changes, as well as autoregressive and cross-lagged effects. Because the ALT accounts for prior levels of both variables when examining associations between initial levels of a variable and mean-level change in a second variable, stronger conclusions regarding longitudinal associations can be drawn using the ALT. The extended LCS model however estimates continuous developmental processes (mean-level changes) as well as proportional change (change that is dependent on the level of personality or life satisfaction at the preceding time point, McArdle, 2009; Young, Furman, & Laursen, 2010). The LCS additionally estimates autoregressive and cross-lagged effects. The drawback of the extended LCS model is that it is complex but nevertheless offers the ability to study the association between intraindividual changes (represented by proportional changes) in one variable and subsequent intraindividual changes in a second variable. This can be useful as models which establish temporal precedence may be susceptible to omitted variable bias since person-specific variables (such as ethnicity or genetic composition or unobserved heterogeneous factors) that may be associated with the variables of interest may not be controlled for appropriately. At least four waves of data are required to apply advanced models such as the ALT and LCS, though more than four waves would be preferable.

In this study we only used data from assessment periods with equal time intervals (Voelkle & Oud, 2015). This is because a focus of the current study was to examine the constant change process. This is change which is assumed to be constant over time and can only be appropriately estimated when there are equal time intervals between assessment periods (Ghisletta & McArdle, 2012). Although using variables that force model constraints, such as phantom variables, can be used to account for unequal time intervals, such an approach can be problematic. This is because with increasing numbers of unequal time intervals, the number of phantom variables required can make the approach unfeasible. Further, the use of phantom variables does not account for heterogeneity in time intervals across intervals and therefore is not suitable for designs where time intervals vary across individuals as well as assessment waves, as is the case in the LISS dataset. This highlights some limitations of the approach: It is not uncommon for studies to have unequal time intervals between assessment periods, thus the utility of the approach may be limited. Further, if only participants who have equal time intervals are included in a study there may be bias owing to nonrandom attrition and variability in responding to assessments over time. However, by using a full information maximum likelihood estimation approach to account for missing data, as we did here, data from all participants who provided data at any of these assessment periods can be used to reduce bias.

## Conclusion

A number of structural equation models are available to examine change in one variable or the longitudinal association between two variables. Each of these models make different assumptions regarding the nature of change and must be interpreted differently. With the availability of repeated measures at three or more time points, more advanced structural equation models can be applied, which account for the complex dynamic nature of change processes and improve our understanding of the nature of the dynamic relation between two or more variables. The choice of model should be determined by theories of change processes in the variables being studied.

## Figures and Tables

**Table 1 tbl1:** Summary of Characteristics of Models and Findings From Previous Studies

Characteristic	Latent growth model	Autoregressive cross-lagged model	Autoregressive latent trajectory model	Latent change score model (including proportional change scores)
Acronym	LGM	ARCL	ALT	LCS
Effects tested				
Baseline correlations	Yes	Yes	Yes	Yes
Mean-level change correlations	Yes	No	Yes	Yes
Subsequent level change correlations	No	Yes	No	No
Autoregressive effect (e.g.: LS level on prior LS level)	No	Yes	Yes	Yes
Cross-lagged effect (e.g.: LS level on prior P level)	No	Yes	Yes	Yes
Prospective effect P level to mean-level LS change	Yes	No	Yes	Yes
Prospective effect LS level to mean-level change	Yes	No	Yes	Yes
Proportional change score to subsequent proportional change score	No	No	No	Yes
Strengths	Models developmental trajectory (mean-level change) + prospective effects	Autoregressive effects, fully prospective	Autoregressive effects + developmental trajectory	Developmental trajectory, proportional change scores, reduced omitted variable bias
Weaknesses	doesn’t model autoregressive effects, not fully prospective, omitted variable bias	doesn’t model developmental trajectory	complex, change between successive waves not explicitly modelled	complex
Previous findings	significant: baseline correlations (all P↔LS), mean-level change correlations (all P↔ LS), prospective effect (N→LS, E→LS, A→LS, C→LS; LS→N, LS→A, LS→C)	significant: baseline correlations (all P↔LS), subsequent level change correlations (all P↔LS), autoregressive effects, cross-lagged effects (N→LS, E→LS, A→LS, C→LS; LS→N, LS→A, LS→C)	No previous research	No previous research
*Note*. LS = life satisfaction; P = personality trait; All p = all personality traits; N = neuroticism; E = extraversion; A = agreeableness; C = conscientiousness.				

**Table 2 tbl2:** Mean and Standard Deviations of Personality and Life Satisfaction Measures at Each Time Period

Variables	Year of measurement			
	2009	2011	2013	2015
Neuroticism	25.79 (6.63)	25.45 (6.72)	25.10 (6.93)	28.34 (8.14)
Extraversion	32.77 (6.32)	32.53 (6.30)	32.37 (6.55)	32.71 (6.44)
Openness	34.85 (4.89)	34.55 (4.90)	34.50 (5.01)	35.83 (5.21)
Agreeableness	38.78 (4.89)	38.47 (4.94)	38.52 (5.07)	38.52 (5.42)
Conscientiousness	36.90 (5.29)	36.88 (5.25)	37.13 (5.32)	35.29 (5.81)
Life satisfaction	25.46 (5.33)	25.27 (5.49)	25.26 (5.53)	25.36 (5.54)

**Table 3 tbl3:** Model Fit Statistics and Parameter Estimates for Latent Growth Model

Model results	Neuroticism		Extraversion		Openness		Agreeableness		Conscientiousness	
Model fit										
CFI/TLI	0.958/0.954		0.958/0.954		0.956/0.952		0.959/0.956		0.954/0.950	
RMSEA	0.026		0.025		0.024		0.023		0.025	
SRMR	0.058		0.065		0.050		0.048		0.045	
Model parameters	β	(*SE*)	β	(*SE*)	β	(*SE*)	β	(*SE*)	β	(*SE*)
Mean-level P change on LS level (**a**)	0.22**	0.03	0.20**	0.03	0.10*	0.03	−0.04	0.03	−0.10*	0.03
Mean-level LS change on P level (**b**)	0.14**	0.05	0.31*	0.05	0.32*	0.10	−0.09*	0.04	−0.07	0.04
Mean-level LS change with LS level (c)	−0.07**	0.03	−0.18**	0.03	−0.18**	0.06	−0.17**	0.03	−0.15**	0.03
Mean-level P change with P level (d)	−0.01**	0.05	0.30	0.19	0.30	0.19	0.04	0.07	−0.03	0.06
P level with LS level (e)	−0.78**	0.08	0.54**	0.05	0.26**	0.04	0.21**	0.02	0.28**	0.02
Mean-level P change with mean-level LS change (f)	−0.58**	0.01	0.13*	0.06	0.08	0.11	0.36**	0.06	0.39**	0.07
*Note*. P = personality; LS = Life satisfaction; CFI = comparative fit index; TLI = Tucker-Lewis index; RMSEA = root mean square error of approximation; SRMR = standardized root mean square residual. Associations are standardized in terms of personality and life satisfaction. Model fit statistics are also presented. Associations are coded to match paths in Figure 1. Key associations of interest are in bold.										
* *p* < .05. ** *p* < .01.										

**Table 4 tbl4:** Model Fit Statistics and Parameter Estimates for Autoregressive Cross-Lagged Model

Model results	Neuroticism		Extraversion		Openness		Agreeableness		Conscientiousness	
Model fit										
CFI/TLI	0.957/0.954		0.957/0.954		0.953/0.949		0.956/0.953		0.951/0.947	
RMSEA	0.026		0.025		0.025		0.024		0.026	
SRMR	0.067		0.053		0.052		0.055		0.054	
Model parameters	β	(*SE*)	β	(*SE*)	β	(*SE*)	β	(*SE*)	β	(*SE*)
P level on prior LS level (**i**)	−0.01	0.01	−0.01	0.01	−0.02*	0.01	−0.02	0.01	−0.02	0.01
LS level on prior P level (**j**)	−0.07**	0.01	0.04*	0.01	0.01	0.01	0.05*	0.01	0.05**	0.01
LS level on prior LS level (g)	0.70**	0.01	0.72**	0.01	0.73**	0.01	0.73**	0.01	0.72**	0.01
P level on prior P level (h)	0.84**	0.01	0.90**	0.01	0.89**	0.01	0.85**	0.02	0.90**	0.01
P level with LS level (k)	−0.47**	0.01	0.26**	0.01	0.14**	0.02	0.16**	0.01	0.23**	0.01
Subsequent P level with subsequent LS level (l)	−0.35**	0.01	0.27**	0.01	0.17**	0.02	0.20**	0.01	0.20**	0.02
*Note*. P = personality; LS = Life satisfaction; CFI = comparative fit index; TLI = Tucker-Lewis index; RMSEA = root mean square error of approximation; SRMR = standardized root mean square residual. Associations are standardized in terms of personality and life satisfaction. Model fit statistics are also presented. Associations are coded to match paths in Figure 3. Key associations of interest are in bold.										
* *p* < .05. ** *p* < .01.										

**Table 5 tbl5:** Model Fit Statistics and Parameter Estimates for Autoregressive Latent Trajectory Model

Model results	Neuroticism		Extraversion		Openness		Agreeableness		Conscientiousness	
Model fit										
CFI/TLI	0.959/0.956		0.960/0.957		0.955/0.951		0.960/0.956		0.954/0.950	
RMSEA	0.025		0.024		0.024		0.023		0.025	
SRMR	0.057		0.044		0.083		0.048		0.045	
Model parameters	β	(*SE*)	β	(*SE*)	β	(*SE*)	β	(*SE*)	β	(*SE*)
Mean-level P change on LS level (**v**)	−0.18**	0.04	0.20*	0.06	0.14**	0.04	0.14*	0.06	0.15*	0.06
Mean-level LS change on P level (**u**)	−0.31**	0.05	0.51**	0.06	0.43**	0.06	0.41**	0.08	0.48	0.08
Mean-level LS change with LS level (q)	−0.10*	0.03	−0.21**	0.03	−0.19**	0.03	−0.23**	0.04	−0.20**	0.04
Mean-level P change with P level (r)	−0.13**	0.03	0.02	0.06	−0.12**	0.03	0.02	0.05	−0.06	0.06
P level on prior LS level (n)	0.12**	0.02	−0.06**	0.01	−0.05**	0.01	−0.06**	0.02	−0.05*	0.01
LS level on prior P level (o)	0.16**	0.02	−0.13**	0.02	−0.10**	0.02	−0.11**	0.02	−0.12**	0.02
LS level on prior LS level (**s**)	0.10**	0.02	0.12**	0.02	0.08**	0.02	0.11**	0.02	0.10**	0.02
P level on prior P level (**t)**	0.05*	0.02	0.01	0.02	−0.12**	0.18	−0.03	0.02	−0.03	0.02
P level with LS level (p)	−0.61**	0.02	0.34**	0.02	0.15**	0.02	0.23**	0.02	0.30**	0.02
Mean-level P change with mean-level LS change (**m**)	−0.92**	0.04	0.96**	0.03	0.54**	0.05	0.65**	0.06	0.64**	0.06
*Note*. P = personality; LS = Life satisfaction; CFI = comparative fit index; TLI = Tucker-Lewis index; RMSEA = root mean square error of approximation; SRMR = standardized root mean square residual. Associations are standardized in terms of personality and life satisfaction. Model fit statistics are also presented. Associations are coded to match paths in Figure 4. Key associations of interest are in bold.										
* *p* < .05. ** *p* < .01.										

**Table 6 tbl6:** Model Fit Statistics and Parameter Estimates for Latent Change Score Model (LCS)

Model results	Neuroticism		Extraversion		Openness		Agreeableness		Conscientiousness	
Model fit										
CFI/TLI	0.924/0.922		0.937/0.935		0.932/0.930		0.938/0.936		0.931/0.929	
RMSEA	0.033		0.030		0.029		0.028		0.030	
SRMR	0.086		0.070		0.078		0.072		0.077	
Model parameters	β	(*SE*)	β	(*SE*)	β	(*SE*)	β	(*SE*)	β	(*SE*)
Mean-level P change with LS level (**ff**)	−0.71**	0.04	0.73**	0.04	0.42**	0.04	0.51**	0.06	0.19*	0.07
Mean-level LS change with P level (**gg**)	−0.62**	0.03	0.40**	0.06	0.28**	0.07	0.32**	0.05	0.45**	0.05
Mean-level LS change on LS level (ee)	0.76	0.01	0.76	0.01	0.76	0.01	0.75	0.01	0.75	0.01
Mean-level P change on P level (hh)	−0.71**	0.04	0.58**	0.03	0.82**	0.02	0.58**	0.03	0.73**	0.02
Proportional P change on prior proportional LS change (**aa**)	−0.25*	0.11	0.79**	0.16	1.02**	0.01	0.42*	0.15	−0.35	0.19
Proportional LS change on prior proportional P change (**bb**)	−0.05	0.03	0.02	0.03	0.10**	0.03	−0.01	0.03	0.10*	0.03
P level with LS level (cc)	−0.48**	0.01	0.28**	0.01	0.14**	0.01	0.17**	0.01	0.24**	0.01
Mean-level P change with mean-level LS change (dd)	−0.91**	0.02	0.82**	0.03	0.54**	0.06	0.69**	0.04	0.50	0.07
*Note*. P = personality; LS = Life satisfaction; CFI = comparative fit index; TLI = Tucker-Lewis index; RMSEA = root mean square error of approximation; SRMR = standardized root mean square residual. Associations are standardized in terms of personality and life satisfaction. Model fit statistics are also presented. Associations are coded to match paths in Figure 2. Key associations of interest are in bold.										
* *p* < .05. ** *p* < .01.										

## Footnotes

1 The personality questionnaire was also administered to all participants in May/June 2008 and to participants who did not respond to prior personality questionnaires during 2010, 2012, and 2014. However, data from these waves were not included in the analysis as the analytic approach used here required equal time intervals between assessment waves.
